# Tensile and High Cycle Fatigue Performance at Room and Elevated Temperatures of Laser Powder Bed Fusion Manufactured Hastelloy X

**DOI:** 10.3390/ma17102248

**Published:** 2024-05-10

**Authors:** Zehui Jiao, Li Zhang, Shuai Huang, Jiaming Zhang, Xudong Li, Yuhuai He, Shengchuan Wu

**Affiliations:** 1 Key Laboratory of Science and Technology on Aeronautical Materials Testing and Evaluation, Aero Engine Corporation of China, Advanced High Temperature Structural Materials Laboratory, Beijing Key Laboratory of Aeronautical Materials Testing and Evaluation, Beijing Institute of Aeronautical Materials, Beijing 100095, China; 2 3D Printing Research and Engineering Technology Center, Beijing Institute of Aeronautical Materials, Beijing 100095, China; 3 State Key Laboratory of Rail Transit Vehicle System, Southwest Jiaotong University, Chengdu 610031, China

**Keywords:** nickel-based superalloy, additive manufacturing, fatigue, tensile, elevated temperature

## Abstract

The application potential of additive manufacturing nickel-based superalloys in aeroengines and gas turbines is extensive, and evaluating their mechanical properties is crucial for promoting the engineering application in load-bearing components. In this study, Hastelloy X alloy was prepared using the laser powder bed fusion process combined with solution heat treatment. The tensile and high cycle fatigue properties were experimentally investigated at room temperature as well as two typical elevated temperatures, 650 °C and 815 °C. It was found that, during elevated-temperature tensile deformation, the alloy exhibits significant serrated flow behavior, primarily observed during the initial stage of plastic deformation at 650 °C but occurring throughout the entire plastic deformation process at 815 °C. Notably, when deformation is small, sawtooth fluctuations are significantly higher at 815 °C compared to 650 °C. Irregular subsurface lack of fusion defects serve as primary sources for fatigue crack initiation in this alloy including both single-source and multi-source initiation mechanisms; moreover, oxidation on fracture surfaces is more prone to occur at elevated temperatures, particularly at 815 °C.

## 1. Introduction

Hastelloy X, a nickel-based superalloy that incorporates solid-solution strengthening elements, such as chromium (Cr) and molybdenum (Mo), exhibits exceptional high-temperature performance, thermal stability, oxidation and corrosion resistance, and excellent fatigue and fracture toughness. Additionally, it possesses excellent hot working formability and weldability properties. Consequently, this alloy finds extensive application in the production of high-temperature components for aeroengines and gas turbines [1,2,3,4]. However, with the development of resource-conserving and environmentally friendly aeroengines and gas turbines, high-temperature components are more inclined to structural lightweight design and integrated manufacturing. Traditional processing methods, such as casting, forging, or welding are constrained by complex manufacturing processes, exorbitant costs, lengthy production cycles, and the wastage of raw materials [5,6,7]. Laser powder bed fusion (LPBF) offers an advanced additive manufacturing (AM) approach that enables integrated fabrication of complex structural parts by sequentially melting metal-powder layers. This technique ensures superior precision in forming with reduced processing timeframes, making it highly suitable for the efficient manufacture of aeroengine and gas-turbine components [8,9].

Fatigue failure is the predominant mode of failure for aeroengine and gas-turbine components that undergo prolonged exposure to high temperatures and complex stress conditions during service. Therefore, significant attention has been devoted by numerous researchers to the fatigue behavior of AM superalloy materials, which are anticipated for utilization in aeroengine applications. Esmaeilizadeh et al. [10] analyzed the high and low cycle fatigue behavior of LPBF-built Hastelloy X at different laser scanning speeds at room temperature (RT). They established a correlation between the fatigue life of LPBF-built Hastelloy X and the process parameters, suggesting that crack initiation occurs earlier due to the higher stress concentration caused by deeper surface valleys at high laser scanning speeds. Esmaeilizadeh et al. [11] further investigated the cyclic deformation and fatigue behavior of LPBF-built Hastelloy X under various strain amplitudes and employed different fatigue models to predict its fatigue life. Palm et al. [12] evaluated the effects of lack of fusion (LOF) defects on the mechanical properties and fatigue properties of LPBF-built Hastelloy X through tensile and high cycle fatigue (HCF) tests, indicating that the defects would significantly reduce the HCF performance of the specimen. Wang [13] studied the four-point bending and tension-tension fatigue properties of LPBF-built Hastelloy X in its as-deposited condition, as well as after hot isostatic pressing (HIP) at RT. The results indicated that the HIP treatment significantly enhanced the fatigue limit of the alloy by consolidating its microstructure and eliminating defects.

In recent years, the high-temperature application environment of nickel-based superalloys has prompted significant research efforts toward investigating the elevated-temperature fatigue behavior of Hastelloy X, leading to several noteworthy reports being published. Karapuzha et al. [14] conducted tensile and HCF tests on solution heat-treated Hastelloy X, which was manufactured using electron-beam powder bed fusion (EPBF) and LPBF processes. The tests were performed at RT and 750 °C, revealing a significant decrease in the alloy’s tensile plasticity at 750 °C due to carbide precipitation and grain-boundary sliding. Additionally, a notable reduction in fatigue strength was observed at 750 °C, with the final failure occurring from surface-initiated fatigue cracks. Moreover, elevated-temperature fatigue testing revealed intergranular cracking caused by grain-boundary precipitates. Lei et al. [15] examined the HCF performances of LPBF-built Hastelloy X specimens with distinct building directions at 400 °C and 600 °C and established a database of the fatigue-test results with material properties of the alloy to build a machine-learning (ML) framework for fatigue life prediction. According to the current state of research, there is insufficient investigation into the fatigue behavior of AM Hastelloy X, particularly under elevated-temperature conditions. In this regard, comprehensive fatigue performance data are still lacking, and the understanding of associated damage mechanisms remains incomplete.

Therefore, this paper presents a systematic experimental investigation of the tensile and HCF performance of the LPBF-built Hastelloy X at room and elevated temperatures in various manufacturing directions. The horizontal (H) and vertical (V) cylindrical bar blanks were fabricated using the LPBF process and subsequently machined into tensile and HCF specimens. The tensile and HCF experiments were conducted at room and elevated temperatures of 650 °C and 815 °C, which represent typical service conditions for combustion-chamber components of an aeroengine made from Hastelloy X. Additionally, the effects of building direction and temperature on these properties were analyzed while also studying the mechanisms underlying HCF failure.

## 2. Materials and Experiments

### 2.1. Materials and Specimens

#### 2.1.1. Specimen Blanks Fabrication

**Figure 1:**
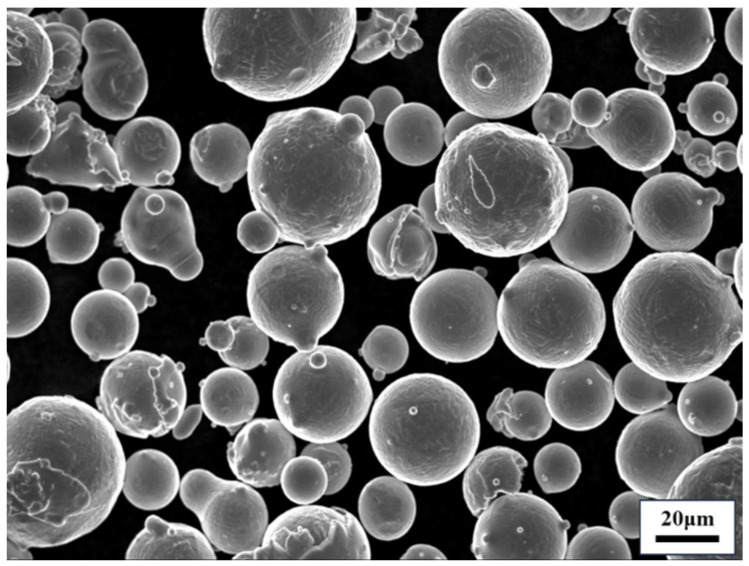
The SEM morphology of the Hastelloy X powder.

**Figure 2:**
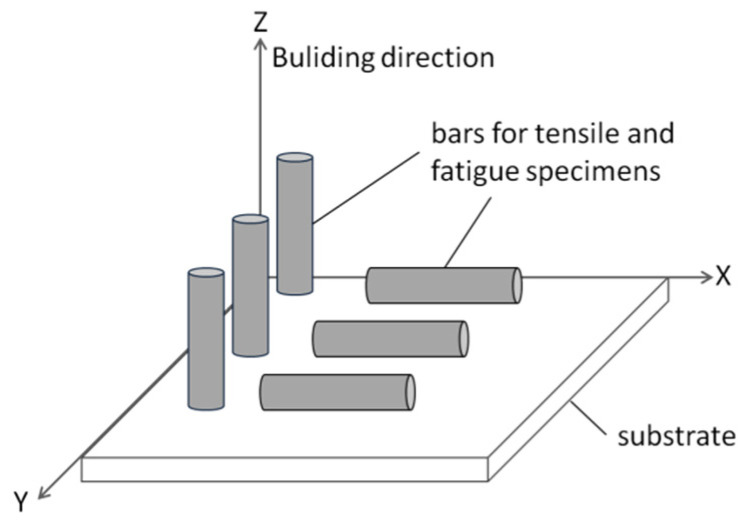
Schematic drawing of cylindrical bars directions [2].

The commercial gas-atomized Hastelloy X powder was supplied by Avimetal Powder Metallurgy Technology Co., Ltd., Beijing, China. The scanning electron microscope (SEM) morphology of the powder is illustrated in Figure 1, revealing highly spherical or nearly spherical powders with a relatively smooth surface and satellite-shaped particles. The particle sizes range from 15 μm to 53 μm, and its chemical composition is shown in Table 1. The LPBF forming equipment utilized was EOS290 (EOS GmbH, Krailling, Germany). To prevent oxidation of Hastelloy X parts during the manufacturing process, a controlled argon environment was maintained within the build chamber with an oxygen concentration below 0.1%. Horizontal and vertical cylindrical bar blanks (Φ15 × 75 mm) were manufactured separately for machining tensile and HCF specimens, respectively. The scanning strategy employed was Stripe. The key process parameters are presented in Table 2. The layout of the bars on the build platform is shown in Figure 2. Following the LPBF process, these Hastelloy X bars underwent a standard solution heat treatment (SHT) at 1177 °C for 1 h in an argon atmosphere and then were cooled in argon at a rate of approximately 40 °C/min.

#### 2.1.2. Metallographic Samples Preparation

Metallographic samples (Φ15 × 5 mm) used for analyzing the microstructure of the YZ plane and XY plane were sectioned using electrical discharge wire cutting from the H-cylindrical bar and V-cylindrical bar of the LPBF-built Hastelloy X separately. Then, they were embedded with epoxy resin and mechanically ground using 400∼2000 mesh abrasive papers and polished using 3 μm and 1 μm water-based polycrystalline diamond suspension, and finally, swab etched with a mixed solution containing 25% HNO3 and 75% HCl for 60 s.

#### 2.1.3. Mechanical Specimens Preparation

**Figure 3:**
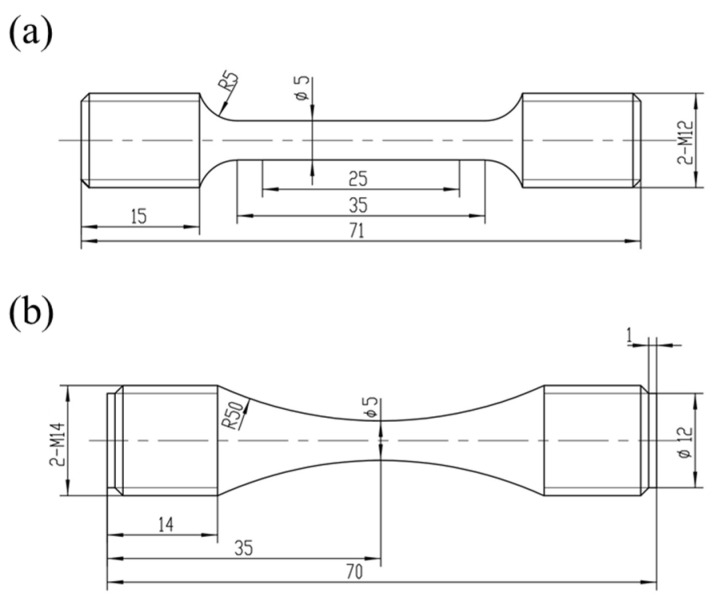
Geometry and dimensions of the (**a**) tensile specimen and (**b**) fatigue specimen (unit: mm) [2].

The tensile and fatigue specimens were precisely machined from cylindrical bars in both directions within a National Aerospace and Defense Contractors Accreditation Program (NADCAP)-accredited machining laboratory. The geometry and dimensions of both types of specimens are depicted in Figure 3. The gauge length of the tensile specimen is 25 mm, with a gauge section diameter of 5 mm and an overall length of 71 mm. The gauge surfaces were ground to achieve a final roughness *R*_a_ value of 0.8 μm. The fatigue specimen exhibits an hourglass shape, featuring a minimum section diameter of 5 mm and an overall length of 70 mm. To minimize machining scratches, the gauge surfaces were manually polished along the longitudinal direction to attain a final roughness *R*_a_ value of 0.4 μm.

### 2.2. Experimental Procedures

#### 2.2.1. Microstructural Observation

The microstructures of both the YZ plane and XY plane were observed using optical microscope (OM) and FEI nano450 Field Emission SEM equipped with an energy-dispersive X-ray spectroscopy (EDXS) system. The grain size rating for both planes was determined using the comparative method of the ASTM E112-13 (2021) standard [16]. The precipitates in the alloy were analyzed using EDXS.

#### 2.2.2. The Tensile Testing Procedures

All tests were performed in the Laboratory of Aeronautical Materials Testing and Evaluation of Beijing Institute of Aeronautical Materials, which holds qualifications from the China National Accreditation Service for Conformity Assessment (CNAS) and NADCAP. The tensile tests at RT were conducted using an Instron 5587 electromechanical testing machine, in accordance with the ASTM E8/E8M-22 standard [17]. The tests at elevated temperatures (650 °C and 815 °C) were performed using an Instron 5892 testing machine, following the ASTM E21-20 standard [18]. The resistance furnace was utilized during testing. Type S thermocouples were employed for temperature measurement, ensuring the deviations between the indicated and nominal test temperatures remained within ±3 °C. Prior to the specimen yield, all tensile tests were carried out at a nominal strain rate of 0.005/min; thereafter, a crosshead rate of 1.75 mm/min was applied once the yield behavior had been recorded. Three specimens were tested under each testing condition, and subsequently, the tensile performance data, including ultimate tensile strength (UTS), yield strength of 0.2% offset (YS), elongation (El), and reduction of area (RoA) were collected. The mean values and standard deviations (SD) were then calculated accordingly.

#### 2.2.3. The HCF Testing Procedures

The HCF tests were conducted using a QBG-50 high-frequency testing machine in accordance with the ASTM E466-21 standard [19] until either specimen failure occurred or 1 × 10^7^ cycles were achieved, at which point the test was considered to have reached “run-out”. The HCF tests were performed under a stress ratio of *R* = −1 and temperatures of RT, 650 °C, and 815 °C. A resistance furnace was utilized for temperature control during the elevated-temperature experiments. Type S thermocouples were employed for temperature measurement, ensuring temperature deviations remained within ±2 °C. A sine wave loading profile was applied at a frequency of approximately 100 Hz. More than 20 specimens were tested under each condition to obtain the *S-N* curve. The generation of the *S-N* curve involved two steps: first by employing the up and down method to determine the fatigue limit and second by selecting four to six maximum stress levels within the medium and shorter life region, with at least two specimens tested under each level. Following completion of the HCF tests, FEI nano 450 Field Emission SEM was utilized to examine the fracture surfaces in order to identify the fracture characteristics and failure mechanisms.

The *S-N* curves were obtained using the three-parameter exponential function, which offers a concise representation for describing the relationship between maximum stress and fatigue life under a constant stress ratio. The function is:

(1) $$N_{f}{\left(\sigma_{max}-\sigma_{0}\right)}^{m}=C$$

This equation can be rewritten in the form:

(2) $$\log N_{f}=B_{1}+B_{2}log(\sigma_{max}-B_{3})$$

where *B*_1_, *B*_2_, and *B*_3_ are material constants. *B*_1_ = log*C*, *B*_2_ = −*m*, and *B*_3_ = *σ*_0_.

## 3. Results and Discussion

### 3.1. Microstructural Analysis

**Figure 4:**
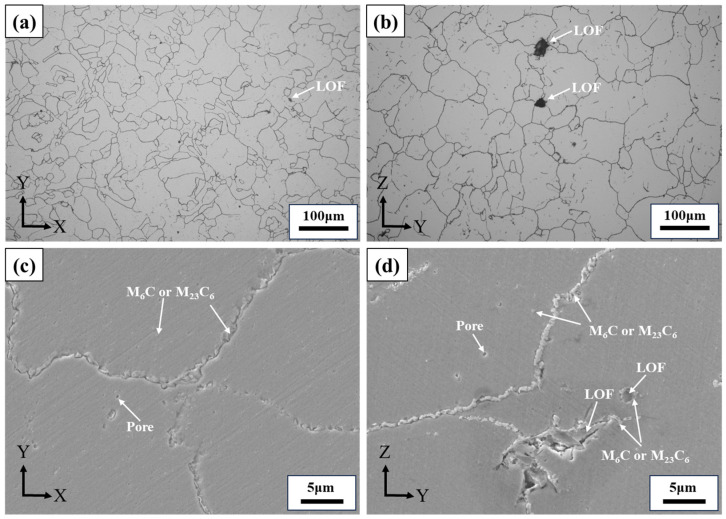
Microstructural characteristics of the LPBF-built Hastelloy X. Optical micrographs showing the morphology of the (**a**) XY plane and (**b**) YZ plane. SEM micrographs displaying carbide precipitates, as well as LOF and pore defects of the (**c**) XY plane and (**d**) YZ plane.

It can be observed that both the XY plane and the YZ plane of the alloy exhibit equiaxed grains with uneven grain size, where the grain size in the YZ plane is larger than that in the XY plane, as shown in Figure 4. The grain size rating of the XY plane corresponds to Grade 5, while the YZ plane corresponds to Grade 4.5. Irregularly shaped LOF defects are visible in both planes, and the quantity and size are greater in the YZ plane than in the XY plane, as seen in Figure 4a,b. A significant number of continuous strip or block carbide precipitates are present at the grain boundaries and the LOF boundaries, along with dispersed granular carbide precipitates within the grains, as seen in Figure 4c,d. The EDXS analysis revealed that these carbides mainly consist of the Mo-rich M_6_C and Cr-rich M_23_C_6_ phases commonly found in AM Hastelloy X alloys. These carbide precipitates at grain boundaries may reduce solid-solution-strengthening elements like Mo and Cr within the γ matrix and diminish its solid-solution strength. In addition, continuous carbides formation can easily lead to cracks along grain boundaries during tensile loading, which may reduce the tensile strength of the material [15,20,21,22,23].

### 3.2. Tensile Properties at Room and Elevated Temperatures

**Figure 5:**
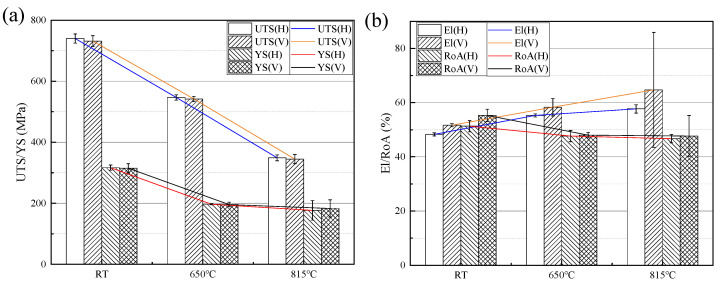
Comparison of the tensile properties at different temperatures of the LPBF-built Hastelloy X: (**a**) UTS and YS; (**b**) El and RoA.

The mean values of tensile properties, including UTS, YS, El, and RoA of the LPBF-built Hastelloy X horizontal and vertical direction specimens at room and elevated temperatures, are present in Table 3 and Figure 5. It is evident that both UTS and YS exhibit a general decreasing trend with an increasing temperature. Specifically, the UTS demonstrates a consistent linear decrease. However, in terms of the YS, there is a significant reduction observed when the temperature increases from RT to 650 °C, but this decline becomes less pronounced as it further rises from 650 °C to 815 °C. Furthermore, the UTS and YS values show no significant variation between the horizontal and vertical directions, with slightly higher values observed in the horizontal direction. According to the Hall-Petch effect [24,25], this consistency can be attributed to the microstructure characteristics depicted in Figure 4 for both directions, which consist of equiaxed grains with a slightly larger grain size rating in the horizontal direction of an approximately 0.5 grade compared to the vertical direction, indicating negligible anisotropy in the tensile properties of the alloy. Additionally, despite Figure 4 illustrating more LOF defects with larger sizes on the YZ plane compared to the XY plane, these factors do not exert significant influence on the tensile strength within a certain range. In terms of plasticity, as shown in Figure 5b, the alloy exhibits good plasticity at room and elevated temperatures, and the El presents a slow rising trend with the increase of temperature. The RoA is observed to decrease at elevated temperatures, potentially due to the inaccuracies in manual measurement. 

Some literatures have reported the tensile property of Hastelloy X obtained through the combined process of AM + HIP [20,26,27]. For instance, reference [20] indicates a UTS of 650 MPa and a YS of 310 MPa in the horizontal direction, as well as a UTS of 575 MPa and a YS of 300 MPa in the vertical direction at RT. Reference [27] demonstrates that the YS in the horizontal direction at 815 °C is 142 MPa. The tensile strength properties observed in this study surpass those previously reported for AM + HIP alloys, as listed in Table 3.

**Figure 6:**
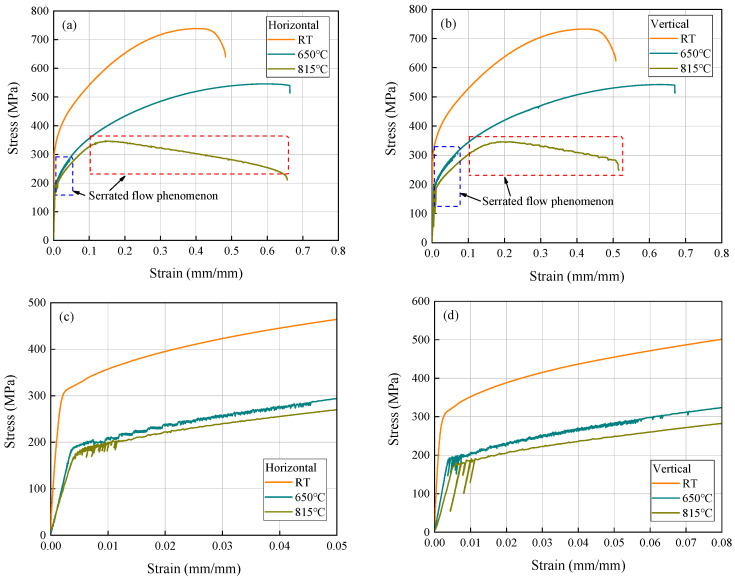
Representative room and elevated temperatures engineering stress-strain curves of the LPBF-built Hastelloy X. (**a**,**b**) Total stress-strain curves of horizontal and vertical directions. (**c**,**d**) The enlarged stress-strain curves in blue frames of (**a**,**b**).

As shown in Figure 5a, the smaller SD values at RT and 650 °C indicate a high level of consistency in the tensile properties data obtained at these two temperatures. However, the SD value for YS at 815 °C is larger. This can be attributed to the fluctuation observed in stress-strain curves during the yield stage, indicating a serrated flow behavior, as depicted in Figure 6. The manifestation of this characteristic is inconspicuous at RT but becomes remarkably pronounced at elevated temperatures. It is primarily observed during the early plastic deformation stage at 650 °C, while it extends throughout the entire plastic deformation stage at 815 °C. Moreover, the fluctuation amplitude is significantly higher than that observed at 650 °C, when the strain is less than approximately 1.2%, but subsequently becomes lower than that of 650 °C. It is precisely because of the more significant serrated flow behavior in the initial yield stage at 815 °C that leads to a larger dispersion of the YS values. This serrated flow phenomenon, also known as the Portevin-Le Chatelier effect, corresponds to the dynamic strain aging (DSA) behavior, resulting from interactions between diffusing solute atoms and mobile dislocations at elevated temperatures [28,29]. Similar serrated flow behavior arising from DSA has been reported in AM nickel-based superalloys [30,31,32]. Furthermore, it is worth mentioning that Figure 5b reveals higher SD values for El and RoA of the vertical direction at 815 °C compared to other temperatures. On one hand, this could be attributed to the manual measurement uncertainties associated with both El and RoA measurements. On the other hand, it may also be related to a limited number of samples (three samples) tested per condition in this study, which might not effectively reflect population-dispersion characteristics. Additionally, an examination of the tensile strength data and stress-strain curves obtained at RT and at 650 °C indicates significant differences between the UTS and the YS of the alloy, exceeding over 400 MPa at RT while approximately reaching around 350 MPa at 650 °C, suggesting excellent deformation strengthening capabilities during the tensile process at these two temperatures. However, 815 °C is obviously different. It is evident from the stress-strain curves that, although the alloy still maintains high YS and El values, the gap between YS and UTS is significantly reduced due to a substantial decrease in UTS.

### 3.3. Fatigue Properties at Room and Elevated Temperatures

**Figure 7:**
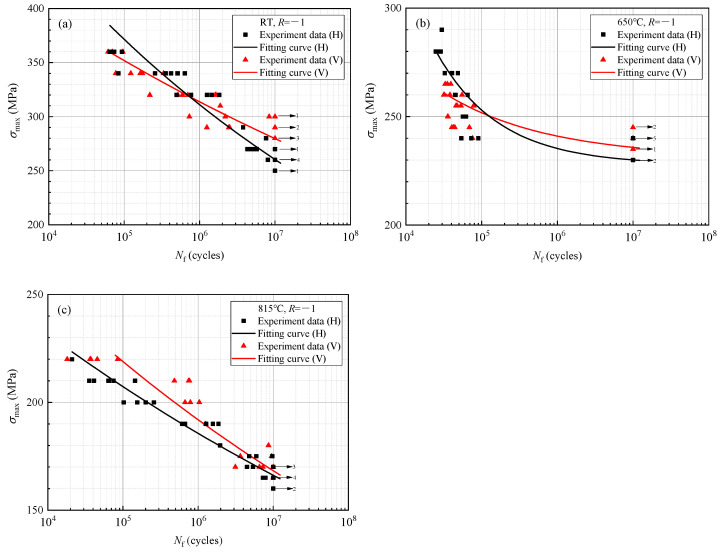
*S-N* curves of different directions of the LPBF-built Hastelloy X at *R* of −1, and temperatures of (**a**) RT, (**b**) 650 °C, and (**c**) 815 °C.

The HCF testing results of the LPBF-built Hastelloy X specimens in both the horizontal and vertical directions, subjected to an *R* of −1 and temperatures of RT, 650 °C, and 815 °C, are presented in Figure 7. The specimens that reached a “run-out” condition at 1 × 10^7^ cycles are indicated by arrowheads, with subsequent numerical values denoting the number of “run-out” specimens at specific stress levels. The *S-N* curves were fitted using the three-parameter exponential function, and the computation values of *B*_1_, *B*_2_, and *B*_3_ for each condition are presented in Table 4.

As shown in Figure 7, the fatigue strength of the LPBF-built Hastelloy X decreases with the increase of cycle times, whether tested at room or elevated temperatures. The horizontal and vertical direction testing results exhibit a high degree of coincidence, indicating the absence of significant anisotropy in the alloy. The fitted *S-N* curves exhibit intersections in both directions at RT and 650 °C, with approximately 7 × 10^5^ cycles at RT and 1.2 × 10^5^ cycles at 650 °C. In the region below this intersection (the short-life (SL) zone), the fatigue strength is slightly higher in the horizontal direction compared to the vertical direction, while in the region above this intersection (the long-life (LL) zone), it is slightly higher in the vertical direction. At 815 °C, there is no intersection between the *S-N* curves for both directions throughout their entire testing-life range, with a slightly higher fatigue strength observed in the vertical direction.

**Figure 8:**
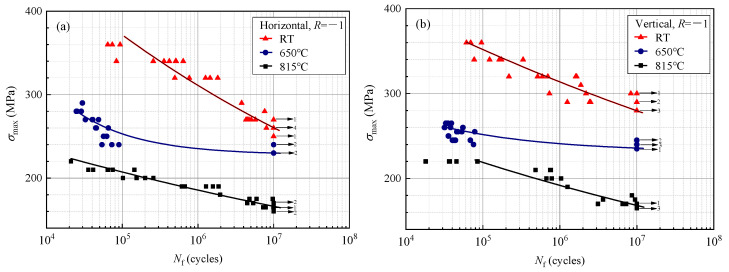
*S-N* curves of (**a**) horizontal direction and (**b**) vertical direction of the LPBF-built Hastelloy X.

The HCF testing results of the LPBF-built Hastelloy X at different temperatures in the horizontal and vertical directions are presented in Figure 8, illustrating a gradual decrease in fatigue resistance with the increasing temperature. It is worth noting that the HCF testing results at 650 °C reveal significant life dispersion under low-stress conditions, especially near the fatigue limit corresponding to 1 × 10^7^ cycles, where there is a gap of over 9 million cycles between the break points and the “run-out” points. This implies that the studied LPBF-built Hastelloy X specimens are prone to rupture earlier around 10^5^ cycles under the fatigue-limit stress level at 650 °C. Therefore, when predicting the fatigue life of these alloy components, it is advisable to employ a relatively conservative life prediction method to ensure safety.

### 3.4. Fractography of the Fatigue Specimens

**Figure 9:**
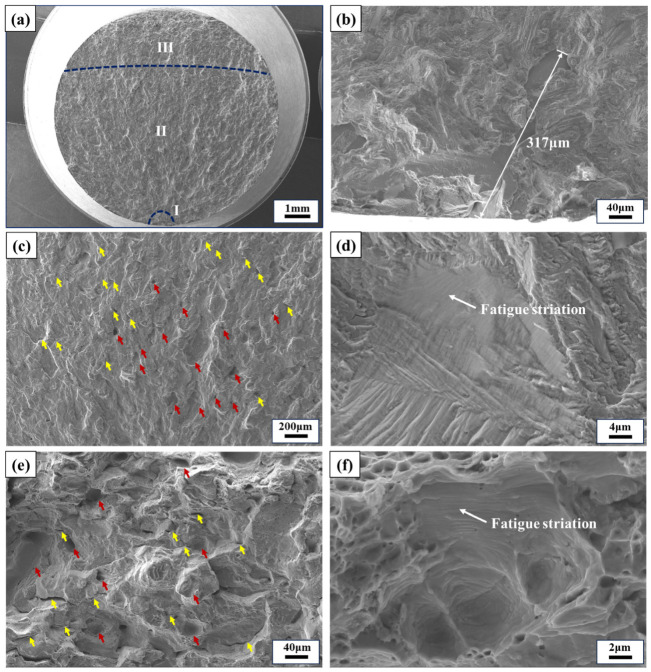
Fracture morphologies of the LPBF-built Hastelloy X specimen in the horizontal direction with conditions of RT, *σ*_max_ of 320 MPa, and *N*_f_ of 494,200 cycles. (**a**) Macroscopic morphology, (**b**) the LOF defect in the crack-initiation region, (**c**) the crack-propagation region, (**d**) fatigue striations in the crack-propagation region, (**e**) the final fracture region, and (**f**) fatigue striations and dimples in the final fracture region.

**Figure 10:**
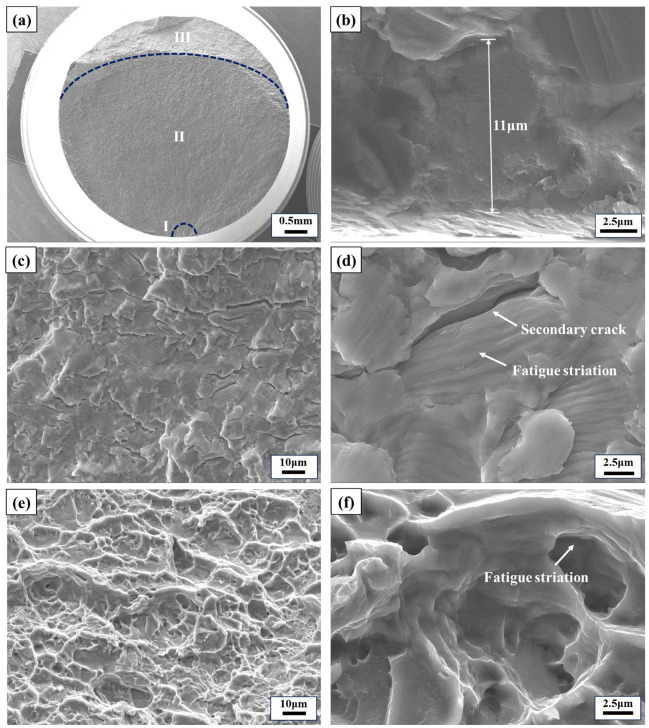
Fracture morphologies of the LPBF-built Hastelloy X specimen in the vertical direction with conditions of RT, *σ*_max_ of 320 MPa, and *N*_f_ of 1,651,500 cycles. (**a**) Macroscopic morphology, (**b**) the LOF defect in the crack-initiation region, (**c**) the crack-propagation region, (**d**) fatigue striations and secondary cracks in the crack-propagation region, (**e**) the final fracture region, and (**f**) fatigue striations and dimples in the final fracture region.

**Figure 11:**
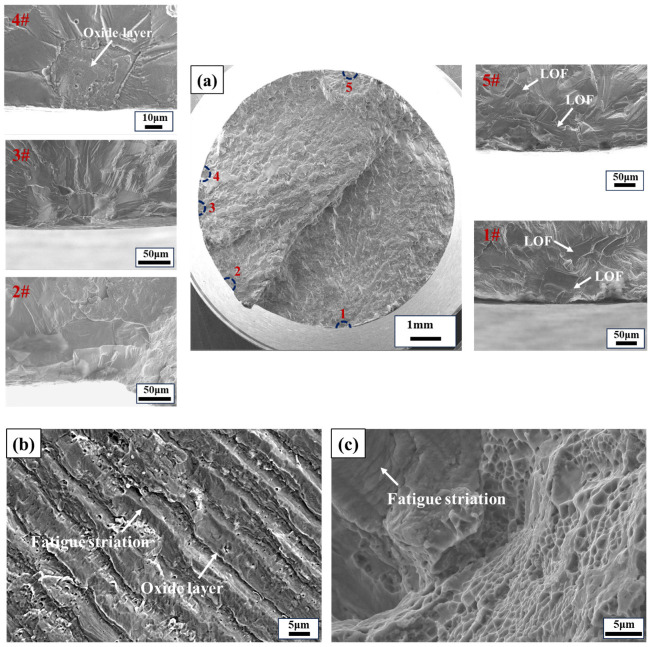
Fracture morphologies of the LPBF-built Hastelloy X specimen in the horizontal direction with conditions of 650 °C, *σ*_max_ of 260 MPa, and *N*_f_ of 45,400 cycles. (**a**) Macroscopic morphology and the LOF defects in the crack-initiation region, (**b**) fatigue striations and oxide layer in the crack-propagation region, and (**c**) fatigue striations and dimples in the final fracture region.

**Figure 12:**
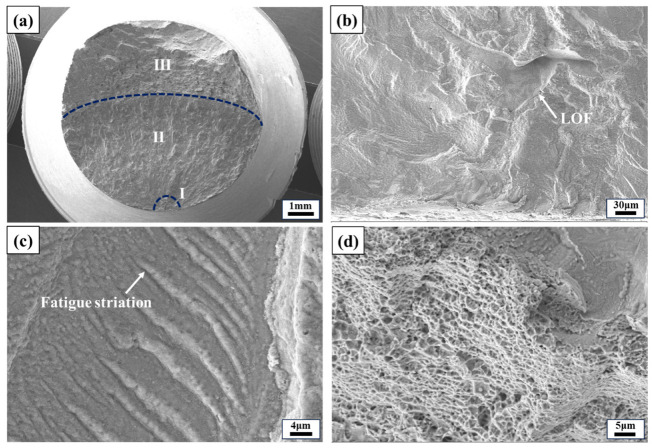
Fracture morphologies of the LPBF-built Hastelloy X specimen in the vertical direction with conditions of 815 °C, *σ*_max_ of 170 MPa, and *N*_f_ of 4,486,100 cycles. (**a**) Macroscopic morphology, (**b**) the LOF defect in the crack-initiation region, (**c**) fatigue striations and oxide layer in the crack-propagation region, and (**d**) dimples in the final fracture region.

The fracture surfaces were analyzed using SEM after conducting HCF tests at room and elevated temperatures, as shown in Figure 9, Figure 10, Figure 11 and Figure 12. It was observed that the main sources of crack initiation were irregular LOF defects located on the subsurface of the specimens, with some specimens having a single LOF defect source while others had two or more. Additionally, there were no significant differences in crack-initiation sources and fracture characteristics between horizontal and vertical direction specimens, which aligns with the results obtained from the microstructure analysis and the fatigue properties testing in both directions. Figure 9 and Figure 10 depict the fracture morphologies of the horizontal and vertical specimens subjected to a test stress of 320 MPa at RT, respectively. The fracture life of the horizontal specimen was recorded as 494,200 cycles (see Figure 9), referred to as the SL specimen, while that of the vertical specimen was recorded as 1,651,500 cycles (see Figure 10), named the LL specimen. The macroscopic morphologies shown in Figure 9a and Figure 10a reveal distinct regions in both specimens, namely crack initiation (region I), crack propagation (region II), and final fracture (region III). In the crack-initiation region, the SL specimen exhibits a larger and irregular LOF defect with the longest axis size of approximately 317 μm (see Figure 9b), while the LL specimen has a smaller LOF defect with the longest axis size of about 11 μm (see Figure 10b). It is widely acknowledged in numerous relevant studies that the shape, size, and location of defects in the crack-initiation region significantly influence the fatigue life of AM alloys, and defects with more irregular shapes, closer proximity to the specimen surface, and larger sizes result in lower fatigue life [33,34,35]. In the crack-propagation region, the SL specimen exhibits numerous LOF defects (indicated by red arrows in Figure 9c), short secondary cracks (indicated by yellow arrows in Figure 9c), and fine fatigue striations (see Figure 9d). The presence of a considerable number of LOF defects within the crack-propagation region was observed across multiple specimens in this study, which not only reflects the existence of internal flaws within the alloy but also suggests that fatigue cracks tend to propagate through these LOF defects. The final fracture region of the SL specimen displays an abundance of LOF defects, secondary cracks, and dimples with varying sizes and depths (see Figure 9e). Moreover, fine fatigue striations are evident within larger dimples (see Figure 9f), indicating that, even in this critical fracture zone, the alloy retains good toughness at RT and maintains a certain lifespan. The crack-propagation region of the LL specimen exhibits minimal LOF defects, while abundant fatigue striations and secondary cracks are observed (see Figure 10c,d). The final fracture region displays numerous dimples of varying sizes and depths (see Figure 10e), with fine fatigue striations also present within larger dimples (see Figure 10f), thereby providing further evidence of the exceptional toughness of the alloy.

**Figure 13:**
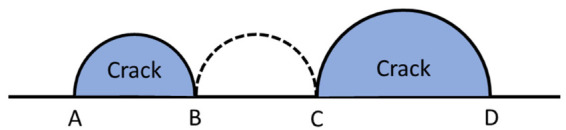
Interaction effect between adjacent cracks [36].

The fracture morphologies of the horizontal direction specimen with conditions of 650 °C, *σ*_max_ of 260 MPa, and *N*_f_ of 45,400 cycles are presented in Figure 11. It can be observed from Figure 11a that the specimen exhibits five crack-initiation sources, all of which are LOF defects. Notably, sources 1# and 5# each have two adjacent defects. Previous research by Murakami [36] has demonstrated that compared to an isolated crack, the interaction between two adjacent cracks tends to increase the stress intensity factor. When a remote tensile stress is applied perpendicular to the crack surfaces, for two adjacent semi-elliptical cracks with different sizes, point C on the larger crack experiences the maximum stress intensity factor, *K*_Imax_, as shown in Figure 13. If there is enough space between the two cracks to insert an additional crack of the same size as the smaller crack, then *K*_Imax_ is approximately equal to that for the larger crack in isolation, thus indicating negligible interaction effects. However, if these cracks are closer to each other than in this case, the *K*_Imax_ at point C increases significantly, and cracks so near to each other are likely to coalesce by fatigue-crack growth in a small number of cycles. The two adjacent LOF defects in the 1# crack source are in close proximity, facilitating their merging into a larger crack and, subsequently, evolving into one of the primary cracks of the specimen. Conversely, the distance between two adjacent LOF defects in the 5# crack source is comparable to that of the smaller LOF defect size. In accordance with Murakami’s research findings, it can be inferred that the interaction in this case can be disregarded, potentially explaining why the 5# crack source fails to propagate into a major crack. Figure 11b shows the fatigue striations in the crack-propagation region of the alloy at 650 °C. Compared with RT, obvious oxidation occurs on the surface of the fatigue striations, but the oxide layers are thinner and tend to peel off from the surface. Figure 11c shows the morphology of the final fracture region, where dimples, oxidation, and fatigue striations can be seen, proving that the alloy also maintains a certain fatigue life in the final fracture region at 650 °C.

The fracture morphologies of the vertical direction specimen with conditions of 815 °C, *σ*_max_ of 170 MPa, and *N*_f_ of 4,486,100 cycles are presented in Figure 12. It is evident that the fatigue fracture mode of the alloy at 815 °C exhibits similarities to those observed at RT and 650 °C. However, compared to 650 °C, oxidation occurs in the crack-initiation region, propagation region, and final fracture region of the specimen at 815 °C, with a thicker oxide layer. Furthermore, upon examining the fracture surfaces of other specimens with equivalent lifetimes as those tested at 650 °C, it is apparent that surface oxidation is significantly more severe for fractures occurring at 815 °C than for those observed at lower temperatures. These findings indicate that the LPBF-built Hastelloy X alloy is susceptible to oxidation when exposed to temperatures as high as 815 °C.

## 4. Conclusions

The tensile and HCF performance at room and elevated temperatures of 650 °C and 815 °C for the LPBF-built Hastelloy X were investigated. Additionally, the effects of building direction on tensile and fatigue properties were studied. The conclusions are summarized as follows:

1. The LPBF-built Hastelloy X exhibits pronounced serrated flow behavior during elevated-temperature tensile deformation. At 650 °C, this behavior is primarily observed in the initial stage of plastic deformation, while at 815 °C, it occurs throughout the entire plastic deformation process. When the deformation is small, the amplitude of sawtooth fluctuations at 815 °C is significantly higher than that at 650 °C. As the deformation increases, the fluctuations weaken sharply and become less prominent compared to those observed at 650 °C;
2. The LPBF-built Hastelloy X demonstrates exceptional deformation strengthening capability during the tensile process at RT and 650 °C, whereas this capability is significantly diminished at 815 °C;
3. The main fatigue-crack initiation sources of the LPBF-built Hastelloy X in this study are irregular subsurface LOF defects, including single-source and multi-source initiation. The alloy fracture surface is prone to oxidation at elevated temperatures, especially at 815 °C, the fracture surface is completely oxidized, and a thicker oxide layer is formed.

## Figures and Tables

**Table 1:** Chemical composition of the Hastelloy X powder (wt.%).

Element	Cr	Fe	Mo	W	Mn	Cu	C	Si	S	Ni
Value	21.600	18.700	8.920	2.010	0.005	0.020	0.080	0.100	0.001	Bal.

**Table 2:** Process parameters of LPBF for the Hastelloy X.

Process Parameters	Laser Power (W)	Scanning Speed (mm/s)	Hatching Space (μm)	Layer Thickness (μm)	Rotation Angle between Layers (°)
Value	260	1100	110	40	67

**Table 3:** Tensile properties of the LPBF-built Hastelloy X specimens at different temperatures.

*T* (°C)	Direction	UTS (MPa)	YS (MPa)	El (%)	RoA (%)
RT	Horizontal	740	317	48.3	51.3
	Vertical	732	315	51.7	55.3
650	Horizontal	547	197	55.3	47.7
	Vertical	542	196	58.3	48.0
815	Horizontal	349	177	57.7	46.7
	Vertical	345	183	64.7	47.7

**Table 4:** Constants values of the *S-N* curves fitting the formula of the LPBF-built Hastelloy X under different conditions.

*T* (°C)	Direction	*B*_1_	*B*_2_	*B*_3_	Correlation Coefficient
RT	Horizontal	38.313	−12.963	0	0.951
	Vertical	56.097	−20.066	0	0.914
650	Horizontal	7.663	−1.901	228	0.850
	Vertical	9.173	−3.167	231	0.723
815	Horizontal	53.244	−20.825	0	0.967
	Vertical	45.944	−17.496	0	0.931

## Data Availability

The original contributions presented in the study are included in the article; further inquiries can be directed to the corresponding authors.
